# Alterations in the Plasma and Red Blood Cell Properties in Patients with Varicose Vein: A Pilot Study

**DOI:** 10.1155/2021/5569961

**Published:** 2021-06-30

**Authors:** Lukasz Gwozdzinski, Anna Pieniazek, Joanna Bernasinska-Slomczewska, Pawel Hikisz, Krzysztof Gwozdzinski

**Affiliations:** ^1^Department of Pharmacology and Toxicology, Medical University of Lodz, Lodz, Poland; ^2^Department of Molecular Biophysics, Faculty of Biology and Environmental Protection, University of Lodz, Lodz, Poland

## Abstract

The varicose vein results from the inefficient functioning of the valves in the lower limb veins, making the blood flow slow down and leading to blood stasis and hypoxia. This type of vein dysfunction might be a result of the development of oxidative stress. We compared oxidative stress markers in the plasma and erythrocytes obtained from peripheral veins and varicose veins in the same patients (glutathione, nonenzymatic antioxidant capacity (NEAC), catalase (CAT) and acetylcholinesterase (AChE) activity, thiols, thiobarbituric acid-reactive substance (TBARS), and protein carbonyls). We found a decrease in NEAC in the plasma obtained from the varicose veins compared to the peripheral veins. We detected a decrease in thiols in the plasma, hemolysate, and plasma membranes and increase in protein carbonyl compounds and TBARS levels in the varicose veins. These changes were accompanied by a decrease in CAT and AChE activity. For the first time, our results show changes in the plasma, erythrocyte membrane, and hemolysate protein properties in varicose vein blood in contrast to the plasma and erythrocytes in peripheral vein blood from the same patients. The increased oxidative stress accompanying varicose vein disease might result from the local inefficiency of the antioxidant defense system.

## 1. Introduction

For a long time, chronic venous disease (CVD) and its main clinical phenomenon—varicose veins (VV)—were often neglected and considered an aesthetic problem. Varicose veins are dilated, tortuous veins that make blood flow indolent and stagnant, causing a higher risk of thromboembolic disease. The presence of varicose veins is the most common clinical symptom of chronic venous insufficiency or CVD affecting adult patients. Many genetic, hormonal, and environmental factors trigger the development of venous system diseases, but age and pregnancy have been found to perform the most prominent role in developing varicose veins [1]. The hallmark of varicose veins is the insufficiency of the venous valves; however, the exact cause and molecular mechanisms leading to such a primary dysfunction have not yet been well identified [2]. The main destructive factor is venous hypertension, which may lead to the remodeling of vein walls and vein valves due to an increase in venous pressure, which leads to structural and functional changes in the vein wall. Various studies have hypothesized that the cause of CVD is the trapping of leukocytes owing to the defective valves and oxidative stress in the veins [3–5]. In addition, hypoxia performs a significant role in the physiopathology of varicose veins [6]. In hypoxia, there is a low-oxygen concentration and a high carbon dioxide concentration, which lead to a decline in pH and, consequently, a rise of free iron ions from transferrin. Iron collection causes enhanced catalysis of free radical reactions that produce other reactive oxygen species (ROS) in the activated inflammatory cells present in venous blood, as well as those present in the wall of varicose veins. This leads to the inflammation of the iron-controlled CVD of the lower limbs [3, 7].

It is postulated that the dysfunction of the vascular endothelium might be the result of an excessive generation of ROS, depletion of antioxidant defenses, and the development of oxidative stress [8]. Eventually, the oxidative stress progresses to CVD, creating a vicious circle. Elevated levels of ROS in varicose veins may result in the plasma and blood cell component oxidation, as evidenced by the significant increase in the presence of ROS metabolites in the plasma derived from varicose veins as compared to the levels of ROS in the plasma obtained from healthy donors [5, 9–11]. Other studies have reported changes in the rheological properties of erythrocytes in varicose veins and, therefore, ROS may lead to the oxidation of proteins and lipids within the erythrocytes' structure [12, 13]. Moreover, changes in the fluidity of membrane lipids and conformational changes in the membrane proteins of erythrocytes present in varicose veins have been observed [13]. Despite numerous studies, the mechanisms associated with varicose vein pathogenesis are still not clear.

Our preliminary study aimed to identify the difference in the oxidative stress markers in the plasma, hemolysate proteins, and erythrocyte membrane samples obtained from varicose vein blood and normal peripheral vein blood (control) in the same patients with CVD.

## 2. Materials and Methods

### 2.1. Chemicals

The following chemicals were purchased from Sigma Chemical Co. (St. Louis, MO, USA): 4-iodoacetamide-TEMPO (iodoacetamide spin label; ISL), 4-Amino-TEMPO (tempamine), 2, 4, 6-tripyridyl-s-triazine (TPTZ), o-phthalaldehyde (OPA), xylenol orange, 5-dithiobis-2-nitrobenzoic acid (DTNB), acetylthiocholine iodide, 2, 4-dinitrophenylhydrazine (DNPH), and 2, 2′-dithiodipyridine. Bis-(2, 2, 5, 5-tetramethyl-3-imidazoline-1-oxyl-4-yl) disulfide biradical (•RSSR•) was obtained from Enzo Life Sciences, Inc. (New York, USA). All other reagents of analytical purity were obtained from POCH S.A. (Gliwice, Poland).

### 2.2. Subjects

Blood samples were collected from eight patients (6 male, 2 female; mean age 54.4 ± 10.6 years; mean BMI 27.7 ± 3.0 kg/m^2^) with varicose veins classified as class C_2_E_s_A_3_P_r_ using a comprehensive system classification for chronic venous disorders (CEAP). The highly selected study group of patients did not suffer from any other chronic or acute disease, including inflammatory etiology diseases, and did not use either phlebotropics or drugs that could affect the blood coagulation process. In addition, in the group of patients, the INR (International Normalized Ratio) (1.03 ± 0.13), sodium (134.4 ± 1.0 mmol/L), and potassium (4.55 ± 0.42 mmol/L) levels in the blood were tested.

Blood samples were taken from a varicose vein and from the antecubital vein from the same patients and transferred into heparinized test tubes. Blood samples were centrifuged to separate the plasma (3000 × g for 10 minutes). The erythrocytes were washed three times by centrifugation with phosphate-buffered saline (5 mmol/L, pH 7.4). The procedure was performed at a temperature of 4°C.

All experiments were conducted in accordance with the principles of the Helsinki Declaration and were in accordance with the ethical principles set out in the Belmont Report: Ethical Principles and Guidelines for the Protection of Human Subjects of Research. The test procedure was accepted by the Bioethics Committee of the Medical University of Lodz. All patients agreed to participate in the study by signing an informed consent document.

### 2.3. Erythrocyte Membrane and Hemolysate Preparation and Protein Determination

The erythrocyte membranes were prepared using the method described by Dodge et al. [14]. The concentration of the plasma and membrane proteins was determined spectrophotometrically with the Folin and Ciocalteu's phenol reagent according to the method outlined by Lowry et al. [15]. The method was based on the reaction of Cu^+^, produced by the oxidation of peptide bonds, with Folin–Ciocalteu reagent. The products of this reaction are optically active with a maximum absorption at 750 nm. The amount of protein in each sample was estimated using a calibration curve plotted by taking different concentrations of bovine albumin as a standard.

The hemolysate was prepared from the erythrocytes after they were lysed with cold water at a ratio 1 : 1.5 and vortexed for 10 min. Hemolysate was centrifuged at 16000 × g for 10 minutes, for the separation of erythrocyte membranes. In the obtained hemolysate, the concentration of hemoglobin (Hb) was estimated using Drabkin's method based on the oxidation of hemoglobin to methemoglobin in the presence of alkaline potassium ferricyanide [16]. Methemoglobin reacts with potassium cyanide to optically active (540 mN) stable cyanmethemoglobin. The molar absorption coefficient of hemoglobin was used to calculate the protein concentration in the samples (*ε* = 44 mmol^−1^·L·cm^−1^).

### 2.4. Spin Labeling of Hemolysate Proteins

We have used electron paramagnetic resonance (EPR) spin label, ISL, specific to proteins, which binds to protein-SH residues. The conformational changes of hemoglobin in the hemolysate were estimated by measuring the relative rotational correlation time (*τ*_*c*_) for ISL attached to Hb as described in an earlier report [17]. The hemolysate was labeled with ISL, dissolved in an ethanol solution (50 : 1) and incubated for one hour at room temperature. Unbound spin label was eliminated by dialysis in 20 mmol/L phosphate buffer, pH = 7.4, for 24 hours at 4°C. The *τ*_*c*_ was determined from the EPR spectra according to the formula described in an earlier report [18]:

(1) $$\begin{array}{c} \tau_{c}=\frac{1}{2}kw_{0}\left(\sqrt{\frac{h_{0}}{h_{+1}}}+\sqrt{\frac{h_{0}}{h_{-1}}}-2\right), \end{array}$$

where *h*_+1_ is the low-field line height, *h*_0_ is the mid-field line height, *h*_−1_ is the high-field line height, *w*_0_ is the mid-field line width, and *k* is a constant equal to 1.19 × 10^−9^ s.

The EPR spectra were recorded on a Bruker ESP 300 E spectrometer at room temperature (22 ± 2°C), operating at a microwave frequency of 9.73 GHz. The instrumental settings were as follows: the microwave power was set at 10 mW, the center field was set at 3480 G with a range of 80 G, and the modulation frequency and modulation amplitude were set at 100 kHz and 1.01 G, respectively.

### 2.5. Plasma Viscosity Determination

The plasma viscosity was measured using 4-amino-TEMPO as described by Morse [19]. The spin label in the ethanol solution was added to the plasma and remained unbound (final concentration of 4-amino-TEMPO 1 mmol/L) for 0.5 h at room temperature.

The relative time of the rotational correlation (*τ*_*c*_) was calculated from the EPR spectra on the basis of the following equation [20]:

(2) $$\begin{array}{c} \tau_{c}=kw_{0}\left(\sqrt{\frac{h_{0}}{h_{-1}}}-1\right), \end{array}$$

where *k* is a constant equal to 6.5 × 10^−10^ s.

The plasma viscosity was calculated according to the following formula:

(3) $$\begin{array}{c} \eta =\frac{\tau_{c\left(\mathrm{plasma}\right)}}{\tau_{c\left(H_{2}O\right)}}\eta_{H_{2}O}, \end{array}$$

where *τ*_*c*(plasma)_ is the rotational correlation time for 4-amino-TEMPO in the plasma, *τ*_*c*(H_2_O)_ is the rotational correlation time for 4-amino-TEMPO in water, and *η*_H_2_O_ is the viscosity of water equal to 1 cP.

### 2.6. Catalase Activity

Catalase (CAT) activity in the hemolysate and the plasma was estimated using hydrogen peroxide (H_2_O_2_) as the substrate [21]. The results were reported in units of CAT activity per milligram of Hb or plasma protein (U/mg protein/min), where 1 U of CAT is defined as the amount of enzyme needed to decompose 1 *μ*mol of H_2_O_2_ per minute.

### 2.7. Acetylcholinesterase Activity Determination

The AChE activity in the erythrocyte membranes was measured using the Ellman spectrophotometric method [22]. Data are expressed as *µ*mol/mg protein/minute.

### 2.8. Determination of Total Nonenzymatic Antioxidant Capacity

The NEAC of the plasma and hemolysate was determined using the ferric-tripyridyltriazine (Fe[III]-TPTZ) complex. In this method, ([Fe(III)-TPTZ]) complex is reduced by cellular antioxidants to a ferrous [Fe-(II)] complex [23].

NEAC was also estimated by the reduction of 2, 2-diphenyl-1-picrylhydrazyl (DPPH) [24]. A calibration curve was prepared for both methods using different concentrations (0–1000 *µ*mol/L) of Trolox. The results were expressed as nmol of Trolox equivalents per milligram protein or Hb.

### 2.9. Determination of Glutathione Concentration

The concentration of reduced GSH in the plasma and hemolysate was estimated using the fluorescent method. The reaction of GSH with o-phthalaldehyde led to a fluorescent product. The fluorescence was measured at 365 nm and 430 nm excitation [25]. GSH concentration was read from the calibration curve prepared from various concentrations of GSH and expressed as nmol/mg protein or Hb.

### 2.10. Determination of Thiol Content

The level of thiols in the membrane and plasma proteins was estimated using Ellman's reagent ((5, 5′-dithiobis (2-nitrobenzoic acid); DTNB) [26]. The absorbances were measured at a wavelength of 412 nm.

The thiols level in the hemolysate was determined using the Egwim and Gruber method with 4, 4′-dithiodipyridine [27]. The 2-thiopyridone formed in the reaction of thiols with 4, 4′-dithiodipyridine was measured at 324 nm.

For both methods, a calibration curve was prepared from different concentrations of reduced GSH. Data are expressed as nmol thiol/mg protein or Hb.

### 2.11. Determination of Protein Carbonyl Compound Concentration

The protein carbonyls content in the plasma and membrane was determined with DNPH [28]. The concentration of protein carbonyl compounds was calculated using the millimolar absorption coefficient (*ε* = 22 mmol^−1^·L·cm^−1^) and expressed as nmol/mg protein.

### 2.12. Determination of Thiobarbituric Acid-Reactive Substances

The lipid peroxidation in the plasma was measured using a TBARS assay [29] with modifications by Rice-Evans et al. [30]. The TBARS levels were calculated using the malondialdehyde (MDA) absorption coefficient (*ε* = 156 mmol^−1^·L·cm^−1^) and were expressed as micromoles per milligram protein (*µ*mol/mg protein).

### 2.13. Determination of Peroxides

The peroxides in the plasma membrane were estimated using xylenol orange. In the presence of peroxides, Fe (II) is rapidly oxidized to Fe (III) [31]. The concentration of peroxides was calculated from the calibration curve obtained for different concentrations of H_2_O_2_ as the standard. Data are expressed as mmol/mg Hb.

### 2.14. Statistical Analysis

The obtained results were subjected to a statistical analysis using the Shapiro-Wilk test to determine the regularity of the distribution. The homogeneity of the variance was checked using the Brown-Forsythe test. The results fulfilled the conditions of normality.

The differences were determined with Student's *t*-test paired data. All data were presented as mean ± SD and median and interquartile range (IQR: from lower quartile Q1 to upper quartile Q3).

The statistical analysis was performed using Statistica v. 13.0.

## 3. Results

In this study, we determined the properties of the plasma, erythrocyte membranes, and hemolysate obtained from varicose vein blood and normal peripheral vein blood (control) in the same patients.  Table 1 shows the morphological characteristics of blood taken from the peripheral and varicose veins of the studied patients.

**Figure 1:**
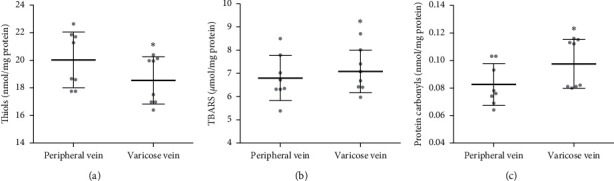
The levels of free thiols (a), TBARS (b), and protein carbonyl compounds (c) in the plasma obtained from peripheral and varicose veins. Data are shown as mean ± range of standard deviation, *n* = 8. ^*∗*^*p* < 0.05 is the significant difference.

The viscosity of the plasma from both sources was determined using a tempamine spin label. There was no difference in the viscosity between the varicose vein and normal vein plasma (Table 2). As shown in Figure 1(a), in the plasma from the varicose vein, the level of free thiols was significantly lower than in the peripheral vein plasma (*p* < .05). On the other hand, we did not find any difference in GSH concentration in the varicose and peripheral vein plasma samples (Table 2). Furthermore, in the varicose vein plasma, a significantly higher level of TBARS and protein carbonyl compounds was observed in comparison to the peripheral vein plasma (Figures 1(b) and 1(c)).

**Figure 2:**
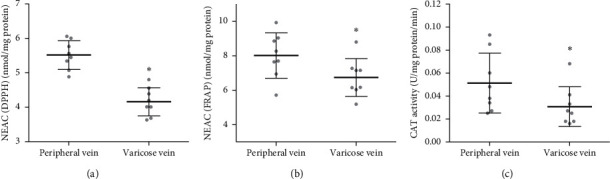
Total nonenzymatic antioxidant capacity in the plasma, determined by (a) DPPH and (b) FRAP methods, and catalase activity (c) in the plasma of the blood samples obtained from peripheral and varicose veins. Data are presented as mean ± range of standard deviation, *n* = 8. ^*∗*^*p* < 0.05 is the significant difference.

To determine the total NEAC of the plasma, two independent methods were used. The changes in the NEAC measured with DPPH showed a significant decrease of NEAC in the plasma of a varicose vein in comparison to the plasma from a peripheral vein (*p* < .05) (Figure 2). The results obtained from the DPPH were similar to those observed using the ferric reducing ability of the plasma FRAP method (Figures 2(a) and 2(b)). Moreover, in the varicose vein plasma, the CAT activity was significantly lower than in the peripheral vein plasma (Figure 2(c)).

**Figure 3:**
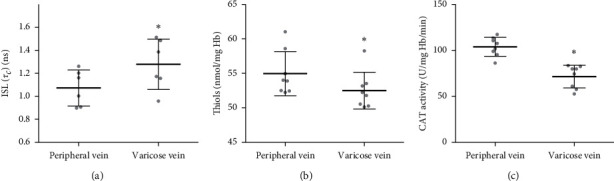
Mobility of ISL labeled hemoglobin (a), the levels of total thiols (b), and catalase activity (c) in the hemolysate obtained from a peripheral vein and a varicose vein. Data are shown as mean ± range of standard deviation, *n* = 8. ^*∗*^*p* < 0.05 is the significant difference.

The EPR spectra of the protein-specific spin labels of maleimide and iodoacetamide used accurately reflect the Hb conformation in peripheral and varicose veins hemolysate. The EPR spectra of spin-labeled Hb with ISL in hemolysate were similar to the spectra of spin-labeled purified hemoglobin. The mobility of ISL attached to Hb from varicose blood was significantly higher compared to the mobility of spin-labeled Hb from a peripheral vein (*p* < 0.05) (Figure 3(a)). This result showed alterations in the structure of this protein in varicose veins compared to peripheral vein blood samples. Moreover, we detected a decrease in the total thiols in the hemolysate from varicose vein blood in comparison to the peripheral vein hemolysates (*p* < .05) (Figure 3(b)). Despite the reduced levels of thiols, the concentration of GSH in erythrocytes from varicose vein and peripheral vein blood did not show any differences (Table 2). Similarly, no significant differences in the total NEAC in the hemolysate from varicose vein and peripheral vein blood were observed (Table 2). Further, the antioxidant enzyme CAT activity was determined in the erythrocyte hemolysate. We observed a significantly lower activity in CAT obtained from varicose vein hemolysate in comparison to the activity determined in peripheral vein hemolysates (*p* < .05) (Figure 3(c)).

**Figure 4:**
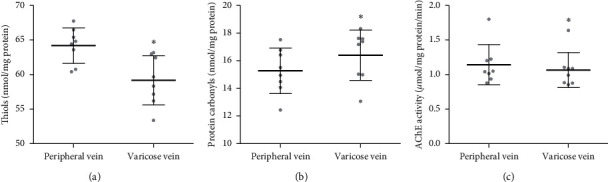
The levels of free thiols (a), protein carbonyl compounds (b), and acetylcholinesterase activity (c) in the erythrocyte membrane obtained from a peripheral vein and a varicose vein. Data are shown as mean ± range of standard deviation, *n* = 8. ^*∗*^*p* < 0.05 is the significant difference.

We also evaluated the oxidative stress parameters in the erythrocyte membranes derived from varicose vein and peripheral vein blood. In the case of erythrocyte plasma membranes, a significant decrease in total thiols from varicose vein blood was found in comparison to peripheral vein blood (*p* < .05) (Figure 4(a)). Moreover, we observed a notable increase in the protein carbonyls concentration in erythrocyte membrane proteins from varicose vein blood in comparison to peripheral vein blood (*p* < .05) (Figure 4(b)). Furthermore, our results reported a reduced AChE activity in varicose vein erythrocyte membranes compared with those of peripheral veins (*p* < .05) (Figure 4(c)). However, the concentration of peroxides was found to be the same in erythrocyte membrane proteins derived from both varicose vein and peripheral vein blood (Table 2).

## 4. Discussion

Varicose veins are enlarged, twisted veins with a diameter of more than 3 mm, and they are the most common lower limb vessel syndrome in humans. Varicose veins result from various factors, such as insufficiency of the vein valves, vessel occlusion, or venous hypertension associated with blood reflux and muscle pump dysfunction [32]. These factors lead to blood stagnation, hypoxia, and the development of chronic inflammation. The hypoxia inducible factor (HIF) and various transcription factors released under hypoxic conditions upregulate the expression of several genes involved in promoting survival under low-oxygen conditions. Hypoxia induces an expression of glycolysis pathway enzyme to generate ATP in an oxygen-independent manner and promoting an expression of vascular endothelial growth factor required for angiogenesis. According to a study, HIF-1a, HIF-2a, and hypoxia perform a crucial role in the pathological process of CVD [33]. More recent studies have shown that the HIF pathway may lead to pathophysiological changes in the varicose vein wall, and hypoxia may contribute to the pathogenesis of VV [34]. However, hypoxia also leads to the increased generation of ROS and, thus, oxidative stress [35, 36]. There are various sources of ROS production, for example, cytoplasmic membranes, endoplasmic reticulum, lysosomes, mitochondria, or peroxisomes [37, 38]. The most efficient route for ROS production in the mitochondrial respiratory chain is during electron transport, with 11 sites producing superoxide and/or hydrogen peroxide [39]. Supposedly, 1% to 3% of oxygen running through the mitochondria is reduced to O_2_^•−^ [40].

In fact, the generation of hydroxyl and alkyl radicals in porcine pulmonary arteries under hypoxic conditions has been demonstrated using spin trapping EPR spectroscopy [41].

Another significant manifestation of CVD is the inflammatory response, due to the interaction of neutrophils and other phagocytic cells, causing the release of various inflammatory cytokines, such as tumor necrosis factors, interleukins (ILs), lymphokines, monokines, and ROS [42]. Activated neutrophils release powerful oxidants that can modify extracellular macromolecules and induce oxidative changes in neighboring cells [43]. Additionally, neutrophils can be activated in the plasma of varicose vein blood and serve as an essential contributing factor in the pathogenesis of primary venous dysfunction [44]. Blood drawn from varicose veins appears to have notably increased concentrations of proinflammatory cytokines IL-6, IL-8, and monocyte chemoattractant protein-1 (MCP-1) in comparison to the blood drawn from the same patient's antecubital vein [45]. Therefore, inflammation in the veins of the lower extremities is more likely than in the veins of the upper extremities. Additionally, it was shown that inflammatory cells, especially leukocytes, perform a key role in both the aging and varicose processes [46]. In addition to being present in vessel walls, ROS can also be found in the plasma due to neutrophil activation.

In our study, we observed lipid and protein peroxidation products, indicated by higher levels of TBARS and protein carbonyl compounds in the plasma of varicose vein patients. The increase in TBARS and protein carbonyl levels was in agreement with the results obtained by Condezo-Hoyos and colleagues, who compared patients with a varicose vein with a group of healthy volunteers [9]. Oxidative damage to proteins and lipids may be associated with a decrease in the level of thiols, as well as a decrease in the level of NEAC in the plasma of varicose veins, which was determined using two independent methods. Additionally, we also found a decrease in CAT activity. It was reported that CAT activity can be inhibited by hydroxyl radicals, superoxide, and H_2_O_2_ but not by organic peroxides [47, 48].

Our previous report showed changes in the erythrocytes from varicose veins in membrane proteins and lipid membrane fluidity [13]. The present study aims to find the reason for these alterations in the internal viscosity of erythrocytes, as well as antioxidant status in internal fluids and membranes. Using covalently bound ISL, we observed an increase in the *τ*_*c*_, reflecting the immobilization of spin label residue. Since the main protein present inside the erythrocytes is Hb, the increased correlation time could suggest conformational state changes in this protein. Moreover, ISL has been reported to bind to *β* globin chain (*β*Cys93) in Hb [49]. It was reported that *β*Cys93 cysteine may influence the redox state of heme iron. Additionally, the oxidative modification of cysteine residue by nitric oxide (NO) leads to the formation of S-nitrosothiol that retains NO bioactivity in the blood [50]. However, irreversible oxidation of this cysteine residue disintegrates the structure of Hb and, consequently, in the release of heme [51]. *β*Cys93 performs a role as a nitric oxide carrier and is also required for the correct oxygenation of tissues and proper cardiovascular function [52]. The increase in *τ*_*c*_ may be attributed to conformational changes in the Hb molecule induced by ROS or its binding to the membrane, since it was shown that mild oxidative stress initiates the binding of redox-active Hb to the membrane [53]. An increase in the correlation time of spin-labeled Hb was also observed after hemodialysis in patients with chronic kidney disease [54], where oxidative stress performs a crucial role in damaging the plasma and its components. In addition to the changes in the Hb structure, a significant decrease in the total thiols and CAT activity in the hemolysate from the varicose vein blood was observed. Free thiol groups (R-SH) are of key importance in protection against oxidative stress because they are susceptible to oxidation by ROS *in vivo* [55]. We also observed a decline in GSH; however, this change was statistically insignificant. No changes in plasma glutathione levels in varicose veins patients in comparison to healthy donors were observed in other studies as well [5].

Our results showed that hypoxic conditions in varicose veins might lead to a decrease of the antioxidant potential of the plasma and lead to the development of oxidative stress, culminating in damage to lipids and proteins. The imbalance between ROS production and removal by antioxidant systems leads to cell malfunction and ultimately to tissue and organ dysfunction [56]. The decreased concentration of total antioxidant status in the plasma and varicose vein wall compared to the control has been reported [5]. Additionally, animal studies under hypoxic conditions have shown an increased ROS production, decreased thiol content, and protein oxidative modification in the form of protein carbonyl content and advanced oxidation protein products, as well as an increase in lipid hydroperoxides and MDA content [57]. The impaired antioxidant defense mechanism and lipid peroxidation in the varicose vein wall in comparison to the control have been also reported [58].

The mechanisms involved in the oxidation of macromolecules lead to damage to the heart muscle. The decreased antioxidant potential was also lowered in patients with chronic heart failure (HF) compared to the control group. On the other hand, in the HF group, oxidative damage to proteins and lipids was observed in erythrocytes, plasma, and saliva, which was associated with an increased ROS production [59]. The major ROS-producing enzyme in a declining heart is xanthine oxidoreductase, and its upregulation contributes to abnormal myocardial hypertrophy, directly contributing to the progression of left ventricular (LV) failure [60]. It has been shown that hypoxia and cytokines, such as TNF-*α*, IFN-*γ*, IL-6, and IL-1, may activate the XOR gene transcription [61, 62].

We also checked the plasma membrane properties for oxidative stress. A significant decrease in the total thiols and an increase in protein carbonyl compounds were detected in the erythrocyte membranes derived from varicose vein blood. Additionally, we determined the AChE activity. AChE present in erythrocyte membranes is an indicator of many processes occurring in the red blood cells, such as changes in the plasma membrane properties, the aging process, inflammation, neurotoxicity disorders, and pesticide poisoning [63]. AChE activity in the erythrocyte membrane from varicose veins was lower in comparison to those of peripheral veins. Our research is in line with work in which oxidative stress might be responsible for a decreased AChE activity in the diaphragms of rats induced with sepsis [64]. Our obtained results suggest oxidative stress in varicose vein blood and increased ROS production in the plasma. Interestingly, oxidative stress is also found in the interior of the erythrocytes. It is possible that superoxide can be generated by damaged Hb or released heme [65]. It is also possible that ROS generated in the plasma can also penetrate the erythrocyte plasma membrane.

Thiol gets oxidized by superoxide radicals or H_2_O_2_ released in the plasma [66, 67]. Both molecules can penetrate through the membrane [68, 69]. Our results align with those reported by Takase et al. [44] and Bujan et al. [46], which state that neutrophils could also be activated in the plasma with the ROS generation. Other ROS can be formed via enzymatic or nonenzymatic pathways, including hypochlorous acid (HOCl) and hypobromous acid (HOBr), generated by neutrophils and eosinophils, respectively, singlet oxygen (^1^O_2_), nitrogen dioxide (•NO_2_), peroxynitrite (ONOO^−^), and carbonate radicals (CO_3_•^−^) [66, 67].

Thus, the present study confirmed that, under conditions of hypoxia and inflammatory response in the blood of varicose veins, activation of phagocytic cells might occur, leading to oxidative damage to the plasma and erythrocyte components.

## 5. Conclusion

The results of the present study showed changes in the plasma and erythrocytes taken from varicose veins in comparison to plasma and erythrocytes taken from peripheral veins in the same patients. It was presented that conditions in the milieu of varicose vein blood lead to oxidative damage of the plasma and erythrocytes and probably other cells in the plasma. Oxidative stress-induced structural changes in erythrocytes may affect their rheological properties, resulting in related complications in microcirculation. Moreover, for the first time, our research showed significant differences in the plasma and red blood cells properties between varicose vein blood and peripheral vein blood in the same patients; however, it is a preliminary study.

## Figures and Tables

**Table 1:** Clinical hematology parameters in patients with venous disease.

Parameter	Peripheral vein	Varicose vein	Statistical significance *p*
WBC (x10^3^/*µ*L)	7.30 ± 1.83	7.97 ± 2,73	0.669
RBC (x10^6^/*µ*L)	4.71 ± 0.56	4.69 ± 0.59	0.985
HGB (g/dL)	14.02 ± 1.34	14.16 ± 1.88	0.673
HCT (%)	40.62 ± 3.32	41.27 ± 4.67	0.612
MCV (fL)	86.67 ± 4.50	88.21 ± 4.92	0.155
MCH (pg)	29.98 ± 1.43	30.21 ± 1.88	0.170
MCHC (g/dL)	34.48 ± 1.06	34.26 ± 1.00	0.849
PLT (x10^3^/*μ*L)	222.70 ± 77.17	191.29 ± 46.28	0.473
RDW-SD (fL)	41.01 ± 3.31	41.79 ± 2.80	0.828
RDW-CV (%)	13.18 ± 0.85	13.24 ± 0.96	0.898
PDW (fL)	11.14 ± 1.31	11.57 ± 2.26	0.237
MPV (fL)	10.21 ± 0.70	10.54 ± 1.03	0.062
NEUT (%)	58.13 ± 11.17	52.54 ± 17.49	0.354
NETU (x10^3^/*µ*L)	4.30 ± 1.49	4.50 ± 2.79	0.870
LYMPH (%)	30.36 ± 9.76	33.70 ± 14.44	0.605
LYMPH (x10^3^/*µ*L)	2.18 ± 0.83	2.37 ± 0.51	0.887
MONOC (%)	8.51 ± 2.06	9.74 ± 2.68	0.052
MONOC (x10^3^/*µ*L)	0.61 ± 0.20	0.73 ± 0.21	0.944
EOS (%)	2,56 ± 1.65	3.56 ± 2.83	0.080
EOS (x 10^3^/µL)	0.18 ± 0.10	0.22 ± 0.12	0.096
BASO (%)	0.44 ± 0.18	0.46 ± 0.21	0.289
BASO (x10^4^/*µ*L)	0.31 ± 0.14	0.33 ± 0.13	0.356

Data are shown as mean ± standard deviation range, *n* = 8, and significance level for Student's *t*-test. Abbreviations: BASO: basophils; EOS: eosinophils; HCT: hematocrit; HGB: hemoglobin concentration; LYMPH: lymphocytes; MCH: mean corpuscular hemoglobin; MCHC: mean corpuscular hemoglobin concentration; MCV: mean corpuscular volume; MONOC: monocytes; MPV: mean platelet volume; NEUT: neutrophils; PDW: platelet distribution width; PLT: platelets; RBC: red blood cells; RDW-CV: red cell distribution width, coefficient of variation; RDW-SD: red cell distribution width, standard deviation; WBC: white blood cells.

**Table 2:** Determined parameters of the plasma, hemolysate, and erythrocytes membrane isolated from blood obtained from peripheral vein and varicose vein blood.

Plasma	Peripheral vein	Varicose vein	Statistical significance
Viscosity (*ƞ*)	1.85 ± 0.04 (1.86; 1.81; 1.87)	1.78 ± 0.07 (1.79; 1.74; 1.82)	n.s.
Reduced GSH (nmol/mg protein)	0.0709 ± 0.007 (0.068; 0.065; 0.078)	0.0692 ± 0.009 (0.069; 0.065; 0.077)	n.s.

*Hemolysate*			
Reduced GSH (nmol/mg Hb)	0.092 ± 0.028 (0.086; 0.071; 0.108)	0.086 ± 0.025 (0.081; 0.072; 0.107)	n.s.
NEAC (FRAP) (nmol/mg Hb)	61.95 ± 4.35 (63.56; 57.35; 65.62)	65.27 ± 5.08 (63.21; 61.94; 68.61)	n.s.

*Erythrocytes membrane*			
Peroxides (mmol/mg protein)	7.17 ± 0.70 (7.14; 6.80; 7.76)	6.98 ± 1.56 (7.06; 5.35; 8.23)	n.s.

Data are shown as mean ± standard deviation range as well as median and quartile 1 and quartile 3 (Me; Q_1_; Q_3_), *n* = 8.

## Data Availability

The datasets used and/or analysed during the current study are available from the corresponding author on reasonable request.

## Abbreviations

AChE: Acetylcholinesterase
CAT: Catalase
CVD: Chronic venous disease
EPR: Electron paramagnetic resonance
GSH: Glutathione
Hb: Hemoglobin
HIF: Hypoxia inducible factor
ILs: Interleukins
ISL: Iodoacetamide spin label
ROS: Reactive oxygen species
TBARS: Thiobarbituric acid-reactive substance.
